# Unmanned Aerial Survey of Fallen Trees in a Deciduous Broadleaved Forest in Eastern Japan

**DOI:** 10.1371/journal.pone.0109881

**Published:** 2014-10-03

**Authors:** Tomoharu Inoue, Shin Nagai, Satoshi Yamashita, Hadi Fadaei, Reiichiro Ishii, Kimiko Okabe, Hisatomo Taki, Yoshiaki Honda, Koji Kajiwara, Rikie Suzuki

**Affiliations:** 1 Department of Environmental Geochemical Cycle Research, Japan Agency for Marine-Earth Science and Technology (JAMSTEC), Yokohama, Japan; 2 Department of Forest Entomology, Forestry and Forest Products Research Institute (FFPRI), Tsukuba, Ibaraki, Japan; 3 Center of Environmental Remote Sensing, Chiba University, Chiba, Japan; NASA Marshall Space Flight Center, United States of America

## Abstract

Since fallen trees are a key factor in biodiversity and biogeochemical cycling, information about their spatial distribution is of use in determining species distribution and nutrient and carbon cycling in forest ecosystems. Ground-based surveys are both time consuming and labour intensive. Remote-sensing technology can reduce these costs. Here, we used high-spatial-resolution aerial photographs (0.5–1.0 cm per pixel) taken from an unmanned aerial vehicle (UAV) to survey fallen trees in a deciduous broadleaved forest in eastern Japan. In nine sub-plots we found a total of 44 fallen trees by ground survey. From the aerial photographs, we identified 80% to 90% of fallen trees that were >30 cm in diameter or >10 m in length, but missed many that were narrower or shorter. This failure may be due to the similarity of fallen trees to trunks and branches of standing trees or masking by standing trees. Views of the same point from different angles may improve the detection rate because they would provide more opportunity to detect fallen trees hidden by standing trees. Our results suggest that UAV surveys will make it possible to monitor the spatial and temporal variations in forest structure and function at lower cost.

## Introduction

Fallen trees are an ecologically relevant indicator of forest biodiversity [1], since they provide habitat for many species, such as small animals (e.g., birds, mammals, and insects) and fungi [2]–[4]. In addition, the decomposition of fallen trees is an important mechanism driving biogeochemical cycles such as nutrient and carbon cycling [5]–[6]. Thus, information about their spatial distribution is of use in indicating species distribution, biodiversity, and biogeochemical cycles in forest ecosystems [7]–[8]. Fallen trees are typically assessed by field surveys [9]. However, because field surveys are time consuming and labour intensive [10], it is expensive to assess the spatial distribution of fallen trees over a wide area. One way to reduce costs is to use remote-sensing technologies such as airborne and satellite imagery (e.g., [10]–[14]). For example, some studies have utilized the remote-sensing technologies for assessment of post-hurricane forest damage (e.g., [15]–[16]).

Recent advances in the technology of unmanned aerial vehicles (UAVs) have made UAVs ideal for remote sensing [17]–[20]. Equipped with sensors such as digital cameras, UAVs can gather aerial photographs with fine spatial and temporal resolution [20]. They also have greater flexibility in flying height and schedule and lower operating costs than manned aircraft [18], [19], [21]. For these reasons, aerial surveys using UAVs have been suggested for low-cost ecological research (e.g., vegetation monitoring and wildlife surveys) [19], [20], [22]–[26].

In this study, we photographed a deciduous broadleaved forest from a UAV, compared the positions of fallen trees identified in the images by eye with those recorded on the ground, and compared the number of the fallen trees detected between the original images and an orthorectified mosaic. The purpose of this study was to show the applicability and limits of this technique.

## Materials and Methods

### Study site

**Figure 1:**
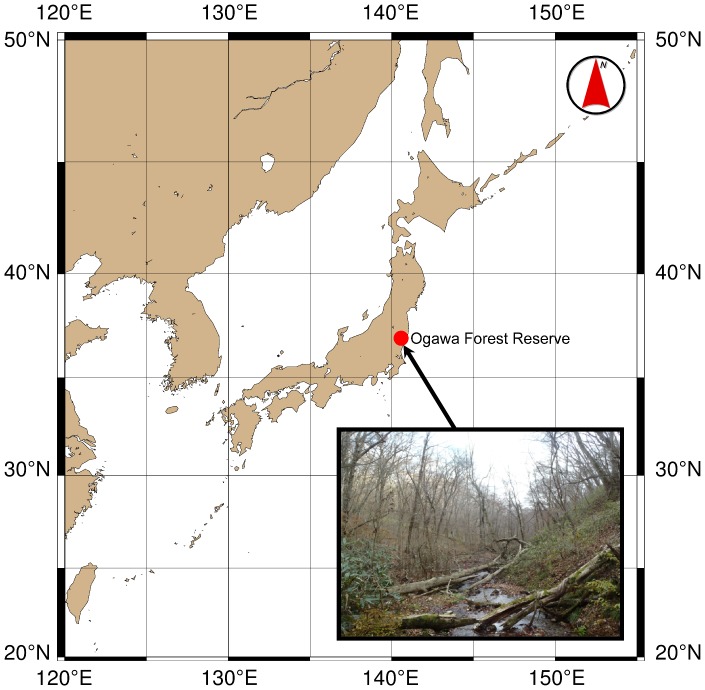
Location of the Ogawa Forest Reserve (OFR).

**Figure 2:**
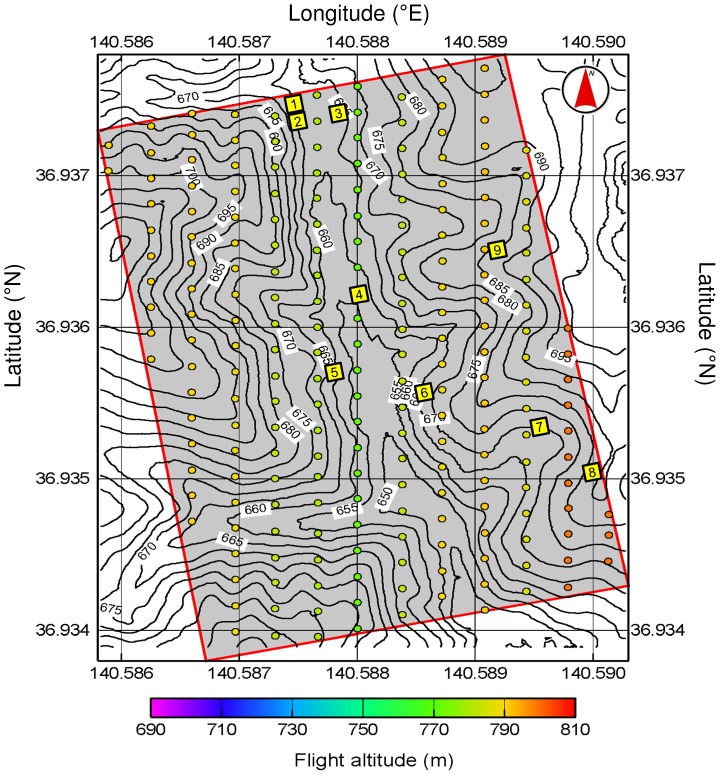
OFR plot (grey area). Yellow squares mark nine sub-plots for ground survey. Circles indicate UAV photograph points. Circle colour indicates flight altitude.

The study was conducted in a 300-m × 200-m plot in the Ogawa Forest Reserve (OFR) in Kitaibaraki, Ibaraki, Japan (36°56′10″N, 140°35′18″E, 650–700 m above sea level; Figs. 1, 2). The forest was dominated by deciduous broadleaved trees such as oak (*Quercus serrata*) and beech (*Fagus japonica* and *F. crenata*) [27], with a patchy distribution of dwarf bamboos on the forest floor [28]. The OFR is described in detail by Nakashizuka and Matsumoto [29]. This study was conducted including a national forest under the permission of the Ibaraki district forest office of the Forestry Agency in Japan.

### Collection of low-altitude aerial photographs from a UAV

**Figure 3:**
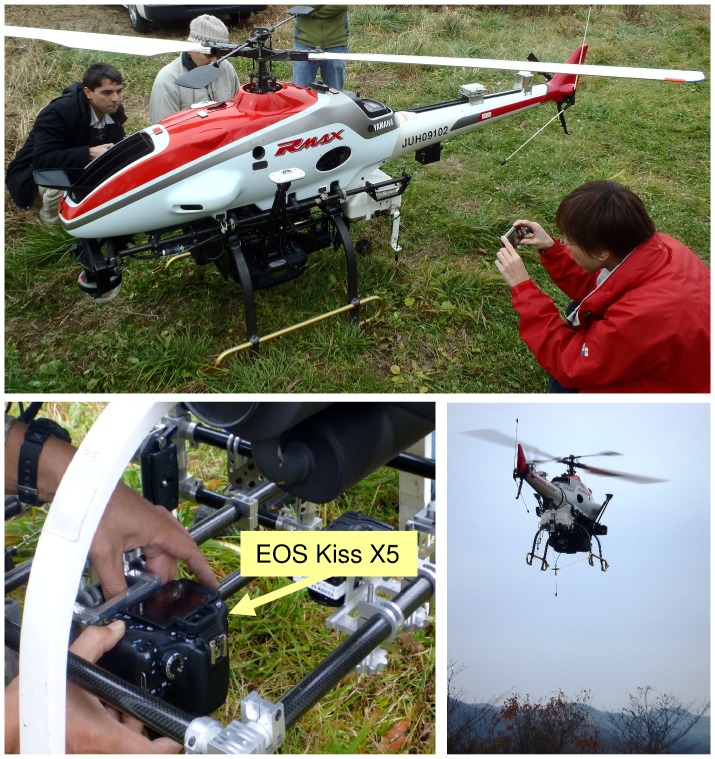
UAV (RMAX-G1) equipped with a digital camera (EOS KISS X5).

On 29 November 2011, when the trees were bare, we flew a UAV (RMAX-G1 helicopter, Yamaha-Motor Co. Ltd., Shizuoka, Japan; Fig. 3; a model commonly used in Japan (e.g., [30]–[32])) over the OFR plot at 30 to 70 m above the ground in a north–south orientation at 3 m s^–1^. A consumer-grade digital camera with a 35-mm lens (EOS Kiss X5, Canon, Tokyo, Japan; image sensor 14.9 mm × 22.3 mm) mounted beneath the UAV pointing straight down took images (5184 × 3456 pixels) every 5 s. The UAV was also equipped with a global positioning system (GPS) detector that recorded altitude, latitude, and longitude every 0.1 s. The GPS position with the timestamp closest to that of each photograph was used as the position of the UAV.

### Generation of a digital elevation model (DEM)

The UAV was also equipped with a laser range finder (LRF; SkEyesBOX MP-1, SkEyes Unlimited Corp., Washington, PA, USA), which assembled a 3D point cloud. We divided the plot (300 m × 200 m) into 1-m × 1-m cells, and the lowest elevation in each cell was used in the creation of a digital elevation model (DEM) of the site.

### Orthorectification and mosaic of aerial photographs

The aerial photographs were orthorectified according to the DEM and the position (longitude and latitude) and attitude (pitch, roll, and heading) of the UAV, and assembled into one mosaic image with the help of “tie points” in overlapping photographs.

### Ground survey

On 31 May 2012 in nine 10-m × 10-m sub-plots (Fig. 2), ground observers recorded the position, stem diameters (each end and midpoint) and length of all fallen trees with a diameter of >5 cm. The ground survey was conducted in leafy season because it was easier to check the tree species in the study site when there were leaves on the trees, as opposed to a leaf-off season.

### Detection of fallen trees in aerial photographs

Fallen trees were identified by eye in the original aerial photographs on a computer monitor. Positions were compared with those of fallen trees mapped in the ground survey and those identified by eye in the orthorectified mosaic.

## Results

**Figure 4:**
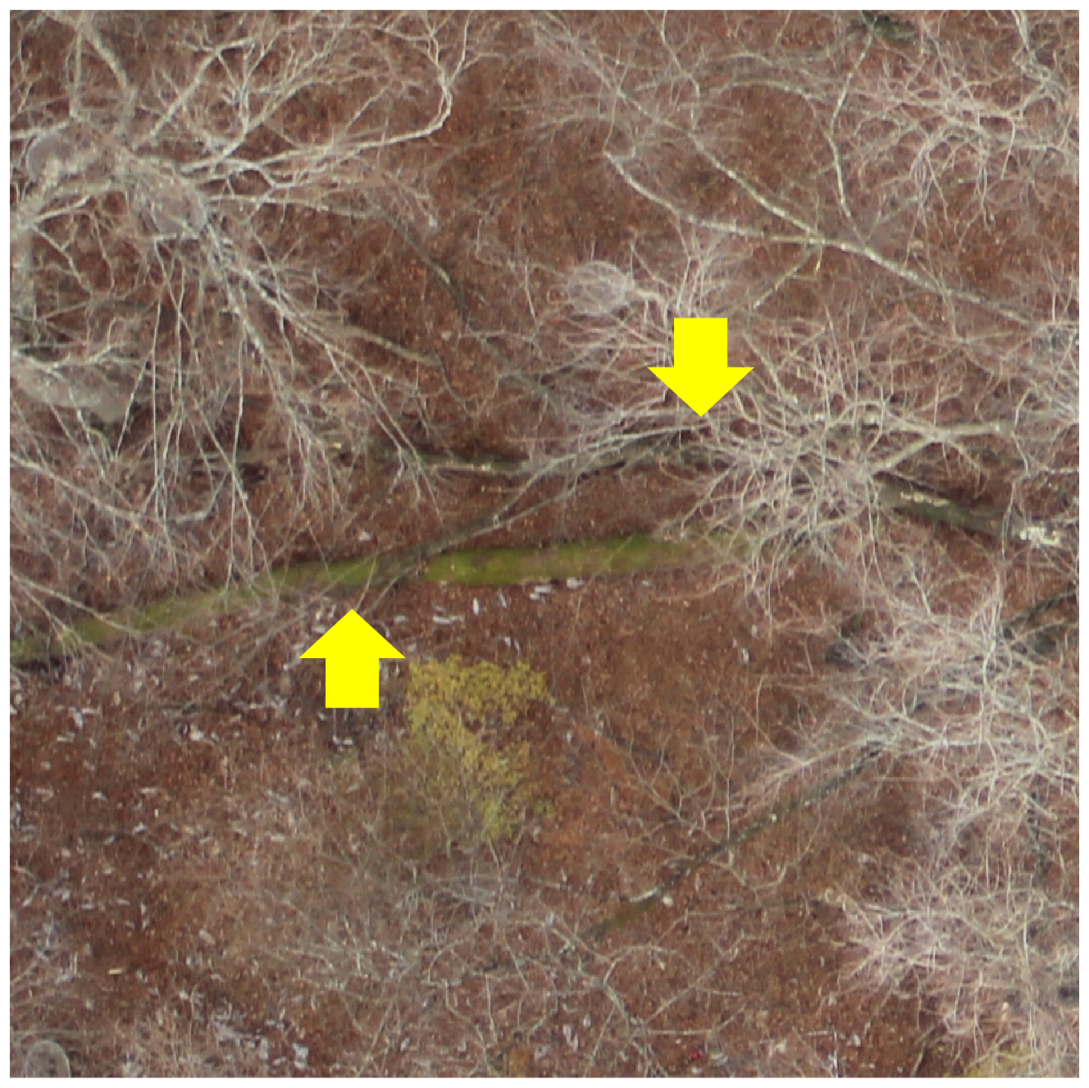
Close-up image of part of the forest floor. Two fallen trees are detectable.

**Figure 5:**
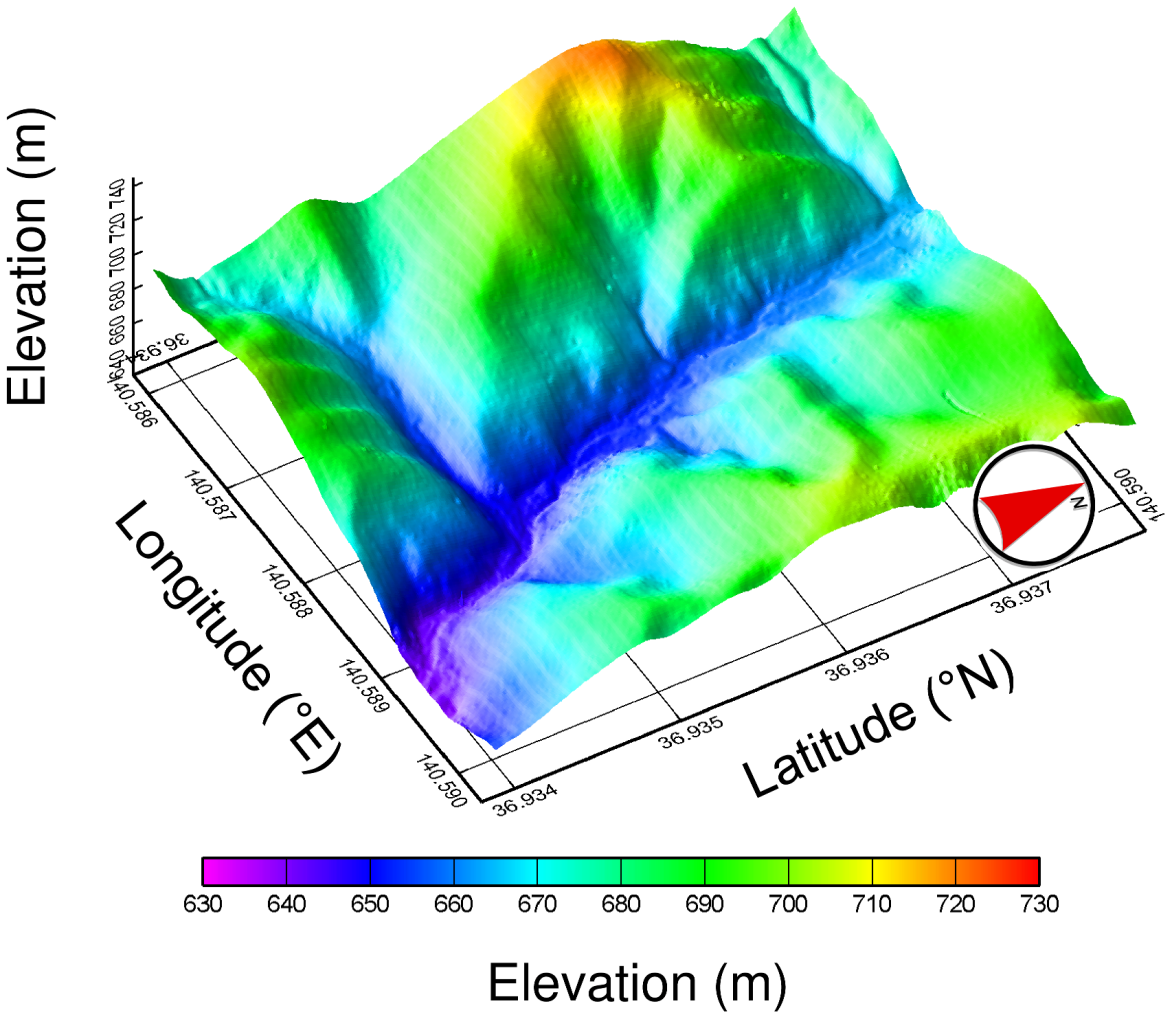
Digital elevation model (DEM) of the OFR plot. This DEM was used for orthorectification of the aerial photographs.

**Figure 6:**
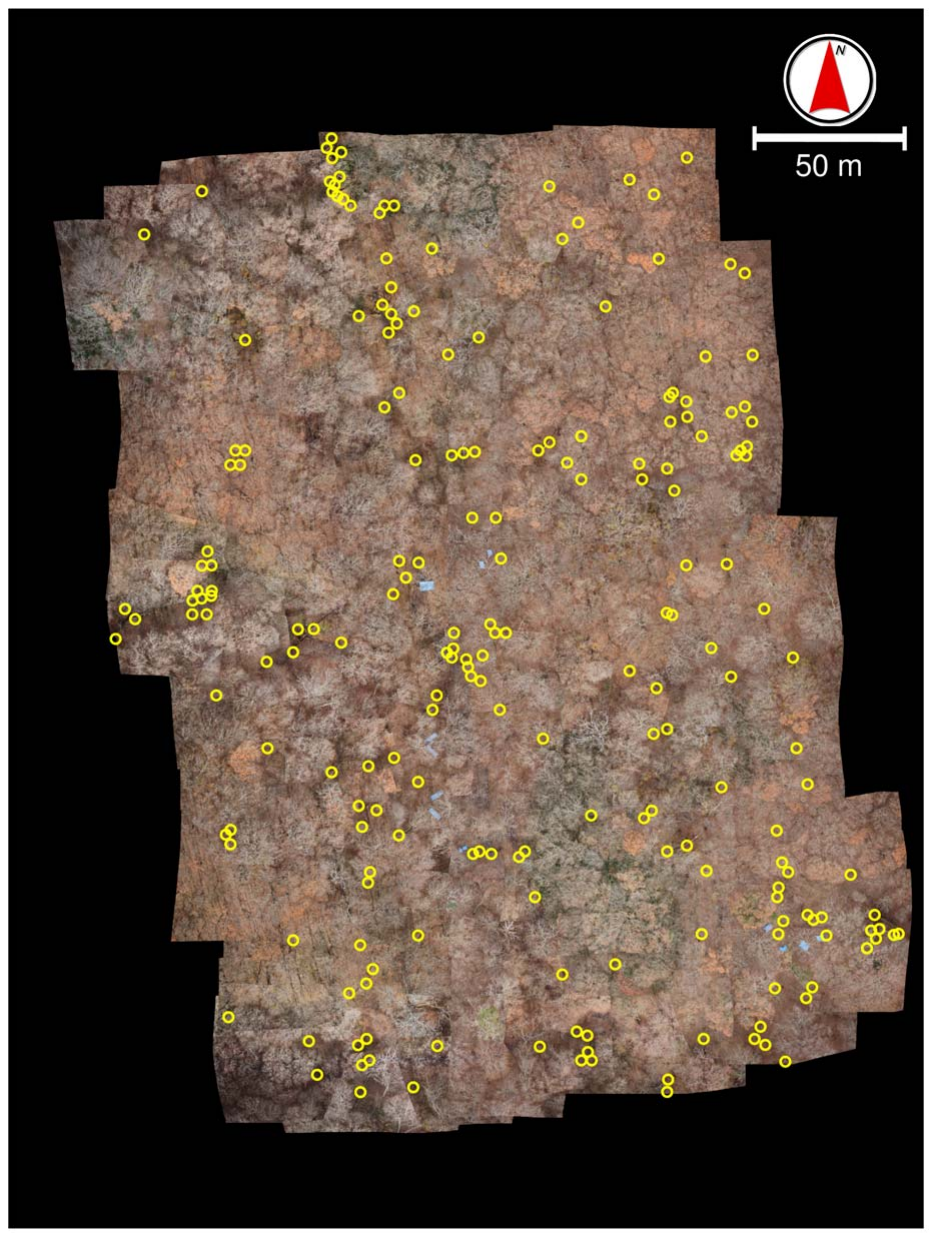
Fallen trees (yellow circles) detected by eye in the orthorectified mosaic.

The OFR plot was covered by 211 aerial photographs (Fig. 2) with a spatial resolution of 0.5 to 1.0 cm per pixel. Since the trees were bare in late November, fallen trees were visible (Fig. 4). A DEM of the plot was generated from a cloud of 5445612 points (Fig. 5). The DEM showed a valley with an elevation range of 640 m (in the south) to 720 m (in the north). From the DEM and the position of the UAV, an orthorectified mosaic image was generated (Fig. 6).

In the ground survey, we found a total of 44 fallen trees in the sub-plots (Table S1). By eye, however, we identified only 11 fallen trees in the sub-plots on the original images (Table S1). We identified 80% to 90% of fallen trees which were >30 cm in diameter or >10 m in length, but few that were thinner or shorter (Tables 1, 2). Over the whole plot, we detected 244 fallen trees on the original photographs and 209 on the orthorectified mosaic (Fig. 6).

**Table 1:** Relationship of maximum diameter between ground-surveyed and visually identified fallen trees.

Maximum diameter* offallen tree (m)	Number of ground-surveyedfallen trees	Number of visually identifiedfallen trees	Identificationrate (%)
0.30≤ *x*	8	7	88
0.20≤ *x* <0.30	7	2	29
0.10≤ *x* <0.20	28	2	7
0.05≤ *x* <0.10	1	0	0

*Maximum of diameters at each end and middle (see Table S1).

**Table 2:** Relationship between lengths of ground-surveyed and visually identified fallen trees.

Length of fallen tree (m)	Number of ground-surveyed fallen trees	Number of visually identified fallen trees	Identification rate (%)
10≤ *x*	9	7	78
5≤ *x* <10	15	3	20
0≤ *x* <5	20	1	5

## Discussion

**Figure 7:**
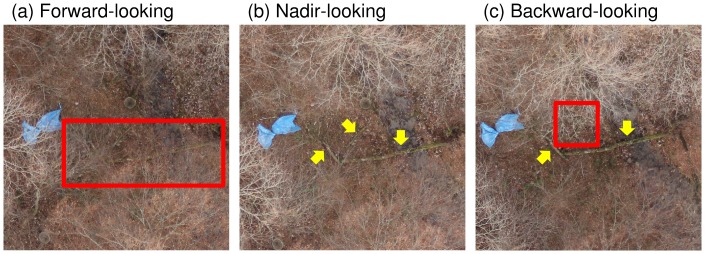
Example of overlapping images of the same point taken from different angles. In this example, the visual detection of three fallen trees from the forward- and backward-looking images may be difficult owing to masking by tree branches (within red boxes). The fallen trees are clearly visible in the nadir-looking image (yellow arrows).

Because fallen trees are generally defined as being >2.5 cm in diameter [2], we assumed that the high spatial resolution of our aerial photographs (0.5–1.0 cm per pixel) would allow us to detect them. However, our results suggest difficulty in identifying narrow or short fallen trees (Tables 1, 2). This failure may be due to the similarity of fallen trees to trunks and branches of standing trees, and masking of fallen trees by the branches of standing trees and forest floor vegetation. Hodgson et al., who surveyed marine mammals by UAV in Australia, reported the usefulness of overlaps between photographs for detecting animals that are masked by sun glitter [24]. For a similar reason, views of the same point from different angles may provide more opportunity to detect fallen trees hidden by standing trees (Fig. 7). The memory capacity of the camera [24] will determine the balance between coverage and overlap. The optimal degree of overlap will also depend on flying speed, flight altitude, and camera specifications (e.g., frames per second).

Another factor contributing to the poor rate of visual identification of fallen trees might be ambiguity in colour. One of the clues we used in detecting fallen trees was the colour of mosses growing on them. Trees with mosses are easy to detect visually, but freshly fallen trees with no mosses might be confused with tree branches or fallen leaves.

The orthorectification and mosaicking of aerial photographs according to the DEM require much labour and time, even for experts, and can also require specialized and expensive software. However, we identified fewer fallen trees from the orthorectified mosaic than from the original photographs. Thus, non-orthorectified photographs of the same point from different angles would allow better identification of fallen trees, at no additional cost, than orthorectified mosaics.

## Conclusions

We showed the applicability of aerial photographs captured from a UAV for the detection of large fallen trees in a deciduous broadleaved forest in eastern Japan. Because much tree death is episodic and irregular [33], high-frequency monitoring at multiple points is necessary for the detection of newly fallen trees and for the understanding of species distribution, biodiversity, and nutrient and carbon cycling in forest ecosystems. UAVs now permit high-frequency monitoring at low cost. This approach has great potential for forest ecology, especially for measuring temporal and spatial variations in forest structure and functioning. Furthermore, as UAVs are advancing and the payload of them is increasing, new sensors for forest monitoring on an UAV will become more common in the future [26]. Installation of a multi-angle photographing system, like a PRISM (Panchromatic Remote-sensing Instrument for Stereo Mapping) on-board the Japanese satellite ALOS (Advanced Land Observing Satellite), on an UAV would potentially help to find more opportunity for detection of fallen trees hidden by standing trees and it may increase the accuracy of fallen tree identification.

## Supporting Information

Table S1 **List of the diameters and lengths of fallen trees in the sub-plots.**
(DOC)
