# Supplementary material for: Unmanned Aerial Survey of Fallen Trees in a Deciduous Broadleaved Forest in Eastern Japan
Source: PLoS One. 2014 Oct 3;9(10):e109881. doi: 10.1371/journal.pone.0109881 (PMC4184894; doi:10.1371/journal.pone.0109881)
Supplement: Table S1 — List of the diameters and lengths of fallen trees in the sub-plots. (DOC) [file pone.0109881.s001.doc]

**Supporting Information**

**Table S1. List of the diameters and lengths of fallen trees in the sub-plots.**

| ID | Sub plot | D_1_ (m) | D_2_ (m) | D_m_ (m) | Length (m) | Visually identified |
| --- | --- | --- | --- | --- | --- | --- |
| 1 | 1 | 0.44 | 0.10 | 0.29 | 20.60 | ○ |
| 2 | 1 | 0.10 | 0.09 | 0.11 | 1.18 | × |
| 3 | 1 | 0.10 | 0.04 | 0.11 | 1.05 | × |
| 4 | 1 | 0.08 | 0.07 | 0.13 | 2.15 | × |
| 5 | 1 | 0.14 | 0.02 | 0.07 | 4.39 | ○ |
| 6 | 1 | 0.09 | 0.08 | 0.10 | 1.00 | × |
| 7 | 2 | 0.40 | 0.12 | 0.15 | 8.90 | ○ |
| 8 | 2 | 0.39 | 0.21 | 0.07 | 17.88 | ○ |
| 9 | 3 | 0.17 | 0.09 | 0.12 | 8.80 | × |
| 10 | 3 | 0.11 | 0.06 | 0.16 | 6.29 | × |
| 11 | 3 | 0.09 | 0.08 | 0.13 | 1.02 | × |
| 12 | 3 | 0.23 | 0.13 | 0.37 | 16.85 | ○ |
| 13 | 3 | 0.13 | 0.08 | 0.22 | 5.29 | × |
| 14 | 4 | 0.22 | 0.06 | 0.15 | 5.47 | × |
| 15 | 4 | 0.28 | 0.09 | 0.21 | 13.33 | ○ |
| 16 | 4 | 0.41 | 0.22 | 0.29 | 6.99 | ○ |
| 17 | 5 | 0.11 | 0.02 | 0.13 | 10.55 | × |
| 18 | 5 | 0.10 | 0.02 | 0.08 | 5.90 | × |
| 19 | 5 | 0.19 | 0.01 | 0.14 | 9.42 | × |
| 20 | 5 | 0.12 | 0.10 | 0.12 | 8.61 | × |
| 21 | 6 | 0.18 | 0.08 | 0.11 | 2.52 | × |
| 22 | 7 | 0.20 | 0.19 | 0.20 | 1.20 | × |
| 23 | 7 | 0.16 | 0.11 | 0.27 | 1.62 | × |
| 24 | 8 | 0.11 | 0.04 | 0.08 | 5.42 | × |
| 25 | 8 | 0.19 | 0.09 | 0.13 | 9.09 | × |
| 26 | 8 | 0.32 | 0.04 | 0.15 | 16.13 | × |
| 27 | 8 | 0.22 | 0.09 | 0.14 | 8.71 | × |
| 28 | 8 | 0.17 | 0.11 | 0.17 | 5.32 | × |
| 29 | 8 | 0.12 | 0.09 | 0.09 | 1.58 | × |
| 30 | 8 | 0.06 | 0.03 | 0.10 | 1.37 | × |
| 31 | 8 | 0.12 | 0.10 | 0.08 | 3.12 | × |
| 32 | 8 | 0.08 | 0.07 | 0.11 | 1.27 | × |
| 33 | 8 | 0.17 | 0.11 | 0.13 | 4.49 | × |
| 34 | 9 | 0.26 | 0.05 | 0.25 | 14.22 | ○ |
| 35 | 9 | 1.01 | 0.42 | 0.58 | 13.79 | ○ |
| 36 | 9 | 0.11 | 0.02 | 0.05 | 4.04 | × |
| 37 | 9 | 0.10 | 0.09 | 0.09 | 2.81 | × |
| 38 | 9 | 0.14 | 0.10 | 0.09 | 1.59 | × |
| 39 | 9 | 0.08 | 0.01 | 0.06 | 8.11 | × |
| 40 | 9 | 0.34 | 0.03 | 0.19 | 14.32 | ○ |
| 41 | 9 | 0.09 | 0.09 | 0.11 | 4.59 | × |
| 42 | 9 | 0.12 | 0.07 | 0.10 | 1.42 | × |
| 43 | 9 | 0.15 | 0.08 | 0.17 | 8.40 | ○ |
| 44 | 9 | 0.09 | 0.08 | 0.10 | 1.48 | × |

ID: Number of fallen tree for identification.

D_1_: Diameter of fallen tree at one end.

D_2_: Diameter of fallen tree at the other end.

D_m_: Diameter of fallen tree at the midpoint.
